# Inulin Functionalized “Giuncata” Cheese as a Source of Prebiotic Fibers

**DOI:** 10.3390/foods12183499

**Published:** 2023-09-20

**Authors:** Diego Romano Perinelli, Agnese Santanatoglia, Giovanni Caprioli, Giulia Bonacucina, Sauro Vittori, Filippo Maggi, Gianni Sagratini

**Affiliations:** Chemistry Interdisciplinary Project (ChIP) Research Center, School of Pharmacy, University of Camerino, Via Madonna delle Carceri 9, 62032 Camerino, MC, Italy; diego.perinelli@unicam.it (D.R.P.); agnese.santanatoglia@unicam.it (A.S.); giovanni.caprioli@unicam.it (G.C.); giulia.bonacucina@unicam.it (G.B.); sauro.vittori@unicam.it (S.V.); gianni.sagratini@unicam.it (G.S.)

**Keywords:** inulin, prebiotic, fresh cheese, functional food, derivatization, UV spectroscopy, water content, rheology, physicochemical characteristics

## Abstract

The development of functional foods in the dairy sector represents a flourishing field of technological research. In this study, an Italian fresh cheese as “giuncata” was enriched with inulin, a dietary fiber, with the aim of developing a product with improved nutritional properties in terms of prebiotic action on intestinal microbiota. An inulin concentration of ~4% *w*/*w* was determined in the fresh cheese after the fortification process, enabling the claim of being a “source of dietary fiber” (inulin > 3 g/100 g) according to the European regulation. The addition of inulin has no effect on the pH of cheese and does not relevantly influence its color as well as the total fat content (fat reduction ~0.61%) in comparison to the control. Mechanical properties of the cheese were also not markedly affected as evidenced from rheological and tensile testing analyses. Indeed, the incorporation of inulin in “giuncata” only exerts a slight “softening effect” resulting in a slightly lower consistency and mechanical resistance in comparison to the control. Overall, this study demonstrates the feasibility of producing a fiber-enriched dairy functional food from a large consumed fresh and soft cheese as “giuncata”.

## 1. Introduction

Inulin is a polysaccharide composed of D-fructose molecules, usually ending with a glucose residue, and occurs as a storage carbohydrate in many plants [1]. For example, it is found in onions (1–5% of fresh weight), garlic (4–12%), bananas (0.2%), and chicory roots (15–20%) [2]. The inulin contained in chicory has a degree of polymerization (DP) of 3 to 60 monosaccharide units, on average about 10; the product of partial enzymatic hydrolysis is oligofructose (OF), which has a DP of 2 to 8 (averagely 4) [3]. This indigestible soluble dietary fiber is now used in an increasing number of foods, including dairy and bakery products, beverages, cereals and granola bars, low-fat spreads, ice cream, and packaged products [4]. Its wide use in the food industry is based on its nutritional and technological properties. As regards the nutritional properties, the prebiotic properties of inulin are important since this fructan can induce a specific change in the composition of the colonic microbiota with beneficial effects on the human host [5]. This increase in beneficial bacteria is found in people of all ages and is associated with several positive physiological effects. These include improved intestinal habits, increased calcium absorption with positive effects on bone health, a reduction in serum lipids with relevance to heart health, a positive effect on satiety with potentially positive consequences for weight management, and a significant effect of increasing resistance to infection and stimulating the immune system [6]. In addition, the technological use of inulin is based on its properties as a sugar substitute (especially in combination with high-intensity sweeteners) [7], as a fat substitute [8], and as a texture modifier [9,10]. Lourencetti et al. conducted experiments with cookies. The partial replacement of fat with inulin in the production of cookies resulted in a decrease in the percentage of total fats in the final product, but also the consistency/texture of the final enrichment product did not change with this substitution [11]. Therefore, the inulin ingredient can be considered as a fat replacer, which is efficient and feasible for the formulation of cookies with good technological and nutraceutical properties. Instead, Mansouripour et al. [12] developed a formulation and produced an acceptable prebiotic ketchup using inulin and GOSs (galactooligosaccharides). For fat replacement in low-fat dairy products, inulin seems to be particularly suitable as it can contribute to a better mouthfeel [13]. Instead, some studies have shown that the addition of long-chain inulin to low-fat yogurt improves its creaminess. Others have shown that this effect also occurs in low-fat cheese [14,15,16,17,18]. When inulin is added to foods at low concentrations, the rheological properties and sensory quality of the product are not greatly affected due to the neutral or slightly sweet taste, and the effects on the viscosity of this ingredient are limited. However, to obtain nutrition and health claims, inulin must be added in amounts greater than 3 g per 100 g or 100 mL [19,20], and a level of 3–8 g per serving should be added to ensure its prebiotic effect. The physicochemical properties of inulin are related to the degree of polymerization [21,22]. The short-chain fraction, oligofructose, is much more soluble and sweeter than native and long-chain inulin and may contribute to improved mouthfeel because its properties are closely related to those of other sugars [23]. Long-chain inulin is less soluble and more viscous than the native product and may act as a texture modifier. Other physicochemical properties influenced by DP include melting transition temperature, the ability to form gels and resulting gel strength, and interaction with other food ingredients such as starch or hydrocolloids [24]. These properties are also important for technical applications of inulin, especially for its ability to modify and improve texture. In the context of the current increase in obesity, there is a strong demand in the food industry for low-calorie, good-tasting foods. Reducing the fat (or sugar) content of a food is, therefore, an important starting point for product development. However, eliminating or reducing fat in foods not only changes the composition and structure, but also the interactions between the different components, resulting in clearly perceptible changes [23]. Highly valued and frequently consumed dairy products can be optimal food matrixes for inulin fortification, due to their impact on the diet of many consumers. Giuncata cheese is an Italian dairy product made from cow’s, sheep’s or goat’s milk. It is a soft and fresh cheese with a texture similar to that of “ricotta” cheese and a higher moisture content that makes it tender. The traditional process for making giuncata cheese is to curdle the milk with rennet or a natural coagulant, and then the curd is collected and placed in molds made of juniper leaves (“giunco” in Italian language), which give the cheese its name. In addition, the juniper leaf molds also give the cheese its unique appearance and flavor. Nowadays for the production of giuncata, firstly pasteurized raw milk is heated and kept at 37 °C. After the addition of salt and rennet, the resulting curd is cut by the cheese maker to help separate the cheese from the whey. The curd is then collected in perforated baskets to allow the whey to drain. This yields the final cheese curd, which can be formed into cylindrical or cuboid shapes using special molds. After molding, the finished cheese is kept in cold water (10 °C) for 2 h before being packaged and stored [25,26].

In the present study, it was chosen to add inulin to the milk, before adding the rennet, to obtain a homogeneous system. The addition of the fiber was quick before the curd forms. As a fresh cheese, giuncata has a soft and creamy consistency, which makes it ideal for spreading, mixing into dishes, or using as a filling for various culinary preparations. For all these interesting reasons, this cheese was chosen for the first time for the development and validation of the inulin fortification design. Therefore, the present study aims to develop an inulin-fortified giuncata and assess whether it can be claimed as a “source of dietary fiber”, without altering its physicochemical and mechanical properties. To our knowledge, this cheese type has never been considered in studies of fortification with inulin, despite its high consumption.

## 2. Materials and Methods

### 2.1. Chemicals and Reagents

The inulin powder (INULIN 90%, 90% of fibers content), obtained from the chicory root (*Cichorium intybus*) was purchased from ACEF (Fiorezuola d’Arda, Italy). The analytical standard for resorcinol (purity ≥ 99%, C_6_H_6_O_2_, molecular weight 110.11 g/mol CAS No. 108-46-3) and the Carrez clarification kit were purchased from Sigma-Aldrich (St. Louis, MO, USA). The extract from the liquid rennet concentrates was produced by Caglificio Clerici spa (Cadorago, Italy). The fresh pasteurized milk was purchased from the company TreValli Cooperlat (Jesi, Italy).

### 2.2. Fortified Giuncata Cheese Manufacturing

As mentioned before in this case, it was chosen to add inulin to the milk, before adding the rennet, in order to obtain a homogeneous system. The addition of inulin was quick and easy before the curd forms. In addition, it does not require the use of machinery other than that used in traditional production, and the subsequent stirring results in a homogeneous product. However, this method did not provide homogeneity of inulin distribution and recoveries in the cheese after coagulation.

**Figure 1:**
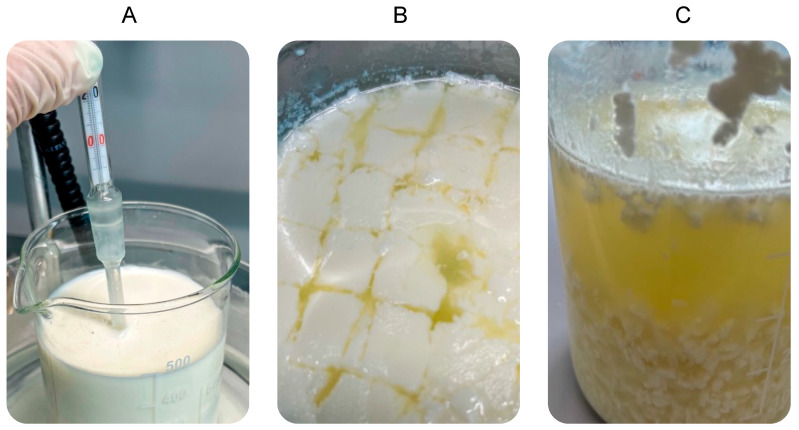
(**A**) Heated fresh milk to 37 °C in a water bath under constant monitoring with a thermometer, and then salt and rennet were added. (**B**) Coagulation. (**C**) Whey removal and separation of whey and curds.

To investigate the inulin recoveries in giuncata cheese and to develop a new functional cheese, the giuncata production process was replicated in the laboratory, in triplicate: first, 500 g of fresh pasteurized milk was weighed, and then the milk was heated to 37 °C in a water bath under constant monitoring with a thermometer. After that, 6 g of salt (1.2 g/100 g of milk), inulin, and finally 0.5 g of rennet were added. After stirring, it took about 30 to 40 min for the milk to curdle properly, and then the resulting gelatinous mass was broken to facilitate the draining of the whey. The drainer used had holes in both the bottom and sides to ensure optimal drainage. After waiting 5 to 10 min to allow the whey to settle, the curd was placed in cheese molds, which gives the giuncata its shape but at the same time allows the whey to settle further (Figure 1). Finally, we cooled the obtained rushes in water at 10 °C for 2 h. Different enrichment experiments were carried out at two concentrations of inulin: 10 g/500 mL and 15 g/500 mL.

### 2.3. Inulin Extraction Procedure

Several extraction tests were preliminarily performed on cheese samples (2 g) fortified with 100 mg of the ingredient starting from the method of [26] to optimize the procedure. Two grams of cheese were weighed and placed in a 100 mL flask, and afterwards 50 mL of boiling water was poured over the sample in the flask. Then, the sample was kept at 85 °C for 10 min, under constant stirring with a magnetic stirrer. Next, the sample was allowed to cool down (20 min) at room temperature and Carrez reagents were added: 5 mL of Carrez reagent I (aqueous solution of K_4_Fe (CN)_6_ × 3H_2_O, 15 g/100 mL) and 5 mL of Carrez reagent II (aqueous solution of Zn (CH_3_COO)_2_ × 2H_2_O, 30 g/100 mL) (Figure S1).

These solutions were used to precipitate the proteins, whose presence interferes with inulin quantification. Finally, the sample was moved to a 50 mL tube, vortexed for 4 min, and then centrifuged at room temperature for 15 min at 5000 rpm. Subsequently, the supernatant was filtered over a 100 mL flask with filter paper and made up to 100 mL with distilled water.

### 2.4. Derivatization and Spectrophotometric Method for Inulin Quantification

The derivatization procedure was firstly performed using 1 mL of standard solution (1 mg/mL inulin solution in distilled water) for the validation of the method. To this solution, 5 mL of a resorcinol reagent (1 mg/mL in EtOH) and 10 mL of a 30% *v*/*v* HCl were added. Subsequently, the samples were then brought to 20 mL through the addition of distilled water, under agitation. Finally, after being covered with foil, they were left in the water bath at 80 °C for 30 min. A temperature of 80 °C was required for the reaction between resorcinol and inulin and the subsequent staining of the sample. Finally, after being cooled down, they were analyzed through a spectrophotometer (Agilent Technologies, Woburn, MA, USA) using 480 nm as the wavelength of quantification.

**Figure 2:**
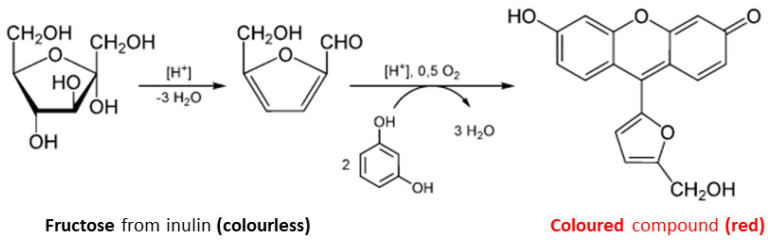
Mechanism of the Seliwanoff test for ketoses. The spectrophotometric method was based on the formation of a colored compound by the interaction of inulin with resorcinol in an acidic environment.

The spectrophotometric method was based on the formation of a colored compound by the interaction of inulin with resorcinol in an acidic environment, as described in the well-known Seliwanoff test for ketoses [27] (Figure 2).

This derivatization reaction is specific for ketoses, and the presence of aldoses does not interfere with inulin analysis. The developed method was validated by studying the linearity, repeatability, detection limits, and quantification of inulin. The repeatability (Table S1) of the analytical method was determined by monitoring the variations in instrumental response (relative standard deviation—RSD %) after analysis of a standard sample (5 ppm) three times in a row on the same day (intraday repeatability) and on three consecutive days (interday repeatability) [28]. The intraday RSD % was 0.2%, while the interday RSD % was 0.7%, confirming very low instrumental variation and repeatability of the analytical method. Subsequently, the developed analytical method was performed to determine inulin concentration in fortified giuncata. A total of 5 mL of the cheese extract containing inulin was diluted up to 50 mL with distilled water and analyzed by UV spectroscopy after derivatization as described above.

### 2.5. Characterization of Inulin-Fortified Giuncata Cheese

#### 2.5.1. Physical–Chemical Composition

A digital pH meter (Mettler Toledo, Columbus, UK) was used to determine pH, while a chroma Meter CR—400 (Konica Minolta^®^, Tokyo, Japan) was used to determine color. The measurement was performed according to the L*a*b* system of the Commission International de l’Eclairage (CIE). In this way, the instrument calculates three parameters simultaneously for each measurement: Brightness (L*); Green to Red component (a*); Blue to Yellow component (b*). By convention, the L* component ranges from 0 (zero brightness, black) to 100 (maximum brightness, a specific white chosen as reference and calibrator). The a* and b* components can take any value except in the cases L* = 100 and L* = 0, where they can only be 0. Positive values of a* indicate a color tending to red, and negative values indicate a color tending to green. Positive values of b* indicate a yellow color, and negative values indicate a blue color.

The fats were extracted according to two methodologies: continuous single-phase extraction and biphasic solvent extraction (Folch extraction). According to the first method, fats extractions were performed using 250 mL of n-hexane as solvent [29]. Extractions were performed using a finely ground 50 g sample of the cheese matrix, which was lyophilized, for one night, and extracted with the selected solvent (n-hexane) in a Soxhlet apparatus (Universal Extractor—Buchi, Mod. E-800, Uster, Switzerland) for 2 h. Then, the extracts were evaporated, and the solvent was removed with rotavapor. At the same time, according to the Folch extraction, 90 mL of chloroform-methanol 2:1 (*v*/*v*) was added to 5 g of ground cheese in a 250 mL beaker and the mixture was extracted with stirring (Ultra-Turrax, IKA, Darmstadt, Germany) for 2 min and then transferred to a separating funnel by washing the beaker walls with a total of 10 mL of solvent mixture [30]. A total of 20 mL of saturated aqueous sodium chloride solution was added; the two phases of the system were shaken, and the organic phase was collected and dried over anhydrous sodium sulphate. Finally, the extracts were evaporated under vacuum and then dried and weighed.

#### 2.5.2. Viscoelastic Properties

Oscillatory measurements (stress sweep test and frequency sweep test) were performed using a rotational rheometer (Kinexus Lab+, Malvern, UK), equipped with a 20 mm plate–plate geometry. For the stress sweep test, rheological moduli (elastic modulus G′ and viscous modulus G″) were recorded at the frequency of 1 Hz in the range of applied stress from 0.1 Pa to 100 Pa at 25 °C. For the frequency sweep test, G′ and G″ were recorded at the applied stress of 1 Pa and in the range of frequencies 0.1–10 Hz. The gap was set at 3 mm. Analyses were performed after one day from the preparation of the cheese in triplicate.

#### 2.5.3. Texture Analysis

Texture analysis on the cheese was carried out using a tensile tester apparatus (5543, Instron, Pianezza, Italy), equipped with a 20 mm diameter cylindrical flat probe. The test speed was 1.0 mm s^−1^, and the depth of penetration was half of the height of the cheese (20 mm). Force/displacement curves were recorded and the parameters “maximum force” (F_max_, N) corresponding to the hardness of the cheese, positive area (N, mm) corresponding to the stiffness of the cheese, negative maximum force (negative F_max_, N), and negative area (N, mm) corresponding to the adhesiveness of the cheese were calculated [31]. Analyses were performed at room temperature after one day from the preparation of the cheese in at least six repetitions.

#### 2.5.4. Thermo Gravimetric (TGA) and Differential Thermal (DTA) Analyses

The weight (%) and heat flow (mW) variations over temperature for the cheese samples were recorded in a nitrogen environment using a simultaneous thermal analyzer STA 6000 (Perkin Elmer Inc., Waltham, MA, USA) at a heating rate of 10 °C/min from 35 °C to 400 °C. The weight loss of samples was determined using the software STA 6000 data analysis (Perkin Elmer Inc., Waltham, MA, USA) using Delta-Y function. Analyses were performed after one day from the preparation of the cheese in triplicate.

#### 2.5.5. Total Water Content

The total water content (%) of the cheese was determined using a moisture analyzer (HE53, Mettler Toledo). A total of 0.7 g of the cheese was heated at 120 °C in an automatic mode until no weight variation was recorded by the instrument. The weight loss of the samples after applying the thermal program was considered as the water content (%) of the samples. Analyses were performed after one day after the preparation of the cheese in triplicate.

### 2.6. Statistical Analysis

All the analyses were performed at least in triplicate (*n* ≥ 3) and data are presented as mean ± standard deviation. Statistical comparisons between control giuncata and inulin-enriched giuncata cheese were performed using Student’s *t*-test and *p* < 0.05 was considered statistically significant.

## 3. Results

### 3.1. Fortified Giuncata Manufactoring

On average, 122–137 g of the cheese was obtained from 500 g of milk, with a yield of 21–24.4%, depending on the amount of inulin added to the milk. Table 1 gives an overview of the inulin content in the cheese at different levels of enrichment (10 and 15 g/500 mL). To confirm the concentration of this ingredient in giuncata cheese, extraction, derivatization, and spectrophotometric analysis were performed according to the methods described previously. The highest inulin contents in giuncata were obtained at a degree of enrichment of 15 g/500 mL of milk (4 g/100 g of the cheese), and the difference with the enrichment of 10 g/500 mL was found to be statistically significant (Table 1). Nutrition and health claims state that a food is a “source of dietary fiber” if the product contains at least 3 g of dietary fiber/100 g [19,20]; therefore, it can be assumed that all enrichment levels tested satisfied this claim. According to this, 15 g/500 mL was chosen as the enrichment inulin concentration in the milk for further studies.

### 3.2. Inulin Fortification and Extraction Procedure

An efficient extraction method was developed to study inulin levels in the fortified cheese (Figure S2). Considering the complexity of the fortified food matrix (giuncata), which is rich in protein (11% *w*/*w*) and fat (15% *w*/*w*), it was necessary to study a selective extraction method for inulin with high recoveries [32].

Among all the preliminary screened procedures, the highest recoveries of inulin from the cheese after derivatization were obtained with the optimized method of [27] performed with some modifications as reported previously (Section 2.3) (Figure S2). This extraction method (Figure S2) achieved recoveries of 79.8 ± 2.8%. The high extraction temperature (85 °C) improved the solubility of inulin in water, thus promoting the extraction.

### 3.3. Study of Giuncata Enrichment

#### 3.3.1. Physical–Chemical Composition

**Figure 3:**
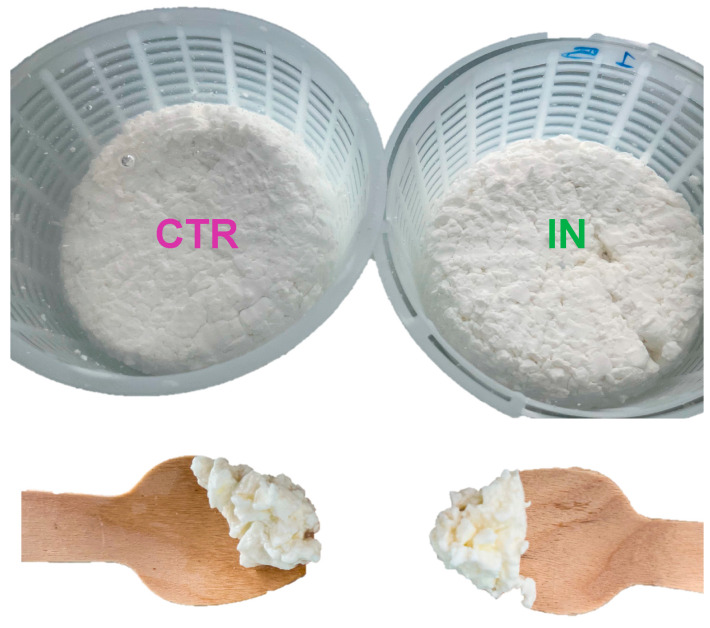
Visual appearance of the cheese Giuncata, produced in the laboratory. **Left** normal (CTR), **right** fortified with inulin (IN).

The enriched giuncata samples (Giuncata IN) (Table 2) were analyzed to study the effects of enrichment on pH, color, and fat content compared to the non-fortified cheese as the control (Giuncata CTR). It was found that the addition of inulin has no effect on the pH of giuncata cheese. Therefore, the measured pH was not significantly different (pH 6.6 vs. pH 6.5) between the Giuncata IN and Giuncata CTR cheeses, as previously reported by [33]. The color of a food product is an important purchase criterion for the consumer. The data regarding color showed that the addition of inulin has no significant effect on the cheese color. However, Giuncata IN cheese tends to show higher brightness (L*: 95.6 ± 0.6) than Giuncata CTR cheese (L*: 93.9 ± 0.2) and a stronger tendency toward blue in functionalized cheese (b*: 7.5 ± 0.6) than in the control, which tends more toward yellow (b*: 7.8 ± 0.7). This positive trend could be explained by the white color of the inulin component. In fact, the white color and less tendency to the yellow color of fresh cheese is perceived by consumers as a quality parameter [17]. Therefore, the enrichment with inulin has a little effect on the color of the cheese but tends to increase the typical white color of giuncata (Figure 3).

The fats were also extracted from Giuncata IN and Giuncata CTR cheeses in order to study the effects of enrichment with inulin on the fat content of fresh cheese and to compare the results (Table 2). Thus, we applied two conventional methods (Folch and Soxhlet extraction), with the combination of different solvents, in order to improve the extraction yield and obtain a more reliable fat profile. The fat content obtained from Soxhlet extraction was 11.72 ± 0.6% and 12.28 ± 0.7% for the Giuncata IN and Giuncata CTR, respectively. The percentage reduction in fat content by Soxhlet was 0.56% (from 12.28% to 11.72%). In the case of Folch extraction, the yields obtained were 11.91 ± 0.2% for the enriched cheese and 12.52 ± 0.3% for the control cheese. The percentage decrease in yield with Folch was 0.61% (from 12.52% to 11.91%). The decrease in lipid content was similar between the two methodologies and these results confirmed the minimal effect of inulin on lipid content, at least for the prepared giuncata cheese.

#### 3.3.2. Viscoelastic Properties of Giuncata Cheese

**Figure 4:**
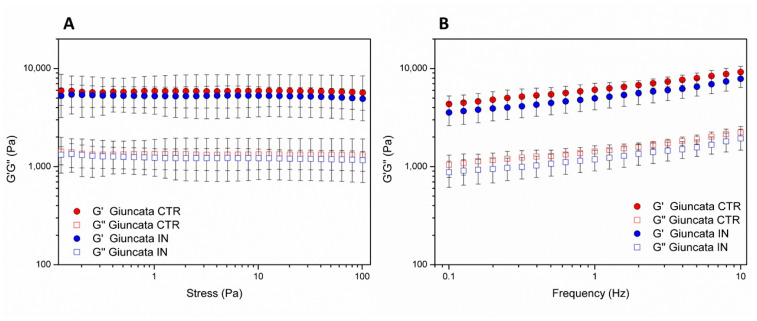
(**A**) Variation of G′ modulus over the applied stress (stress sweep test) and (**B**) variation of G′ and G″ moduli over applied frequency (frequency sweep test) for control giuncata cheese (Giuncata CTR) and inulin-fortified giuncata (Giuncata IN) measured at 25 °C.

The viscoelastic properties of giuncata cheese were assessed by measuring the rheological moduli (G′ and G″) as a function of the applied stress (stress sweep test) and the applied frequency (frequency sweep test). The stress sweep test (Figure 4A) showed the variation of G′ modulus over the applied stress that can be considered as a measure for the consistency of a semi-solid material as giuncata cheese. Specifically, the measured G′ values were almost constant, indicating that the cheese does not destructure over the experimental conditions of applied stress. Moreover, the obtained G′ values were similar between the giuncata control (Giuncata CTR) and inulin-fortified giuncata (Giuncata IN) around 5000–6000 Pa with mean values only slightly lower for Giuncata IN than Giuncata CTR. The frequency sweep test (Figure 4B) highlighted the solid-like behavior of both giuncata cheese, since the G′ values were higher than the G″ values over all the applied frequencies. Indeed, when G′ > G″ the material shows a prevalence of the elastic properties over the viscous ones. Even more, according to the frequency sweep test, it can be noticed that both G′ and G″ moduli are slightly higher for Giuncata CTR than Giuncata IN, confirming the not marked effect of the incorporation of inulin in both the consistency and viscoelastic properties of giuncata cheese. Moreover, both cheeses show a dependency of both moduli over the applied frequency (i.e., the values of both moduli increase over frequency) denoting them as “weak” solid-like samples. This behavior over frequencies has been explained through a not complete relaxation of the network structure of the cheese over the frequency increase [34], and it has been observed for other soft cheese such as mozzarella [35] and others [36,37].

#### 3.3.3. Texture Analysis

**Figure 5:**
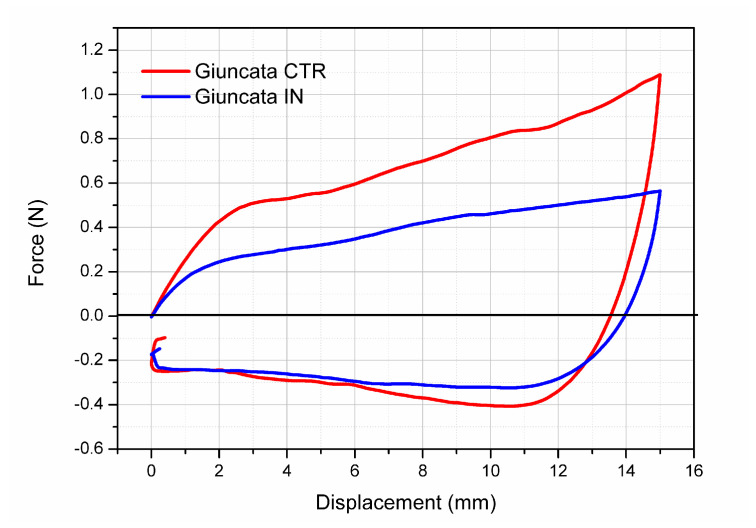
Displacement (mm) vs. force (N) for control giuncata cheese (Giuncata CTR) and inulin-fortified giuncata (Giuncata IN) registered during the penetration of 20 mm flat probe at room temperature.

The mechanical properties of giuncata cheese can be further investigated using texture analysis. Figure 5 shows the displacement vs. force plots obtained from the compression analysis of Giuncata CTR and Giuncata IN both during the penetration of the probe inside the cheese and its release up. For all samples, the maximum force was recorded at the highest penetration (20 mm) of the probe inside the cheese (set as the half distance of the height of the cheese). The increase in the registered force is not linear, but it shows two main slopes. Specifically, a sharper increase in the force was registered in the first 2 mm of compression, related to the elasticity of the material. At any displacement value, the registered force for Giuncata IN was lower than for Giuncata CTR, confirming that as for the consistency (measured by rheology), the mechanical resistance to compression is also affected by the incorporation of inulin into the cheese. These results are comparable to others reported in the literature in which inulin was incorporated into the cheese with the aim to act as a “fat replacer” [33,38]. The softening effects exerted on the cheese by the incorporation of inulin have been supposed to be related to the higher moisture content in relation to the protein content and to the increase in filler volume [39].

The effect of inulin fortification emerges also by considering the calculated texture parameters as compressive force (N), stiffness (N mm) and adhesiveness, which are reported in Table 3.

All these parameters showed a decrease in their value after the incorporation of inulin, for which the statistical comparison was significant for the compressive force (*p* = 0.0065) and stiffness (*p* = 0.0327) but not for adhesiveness (*p* = 0.4428). Compression force, also referred as firmness, is the force required to obtain a certain deformation and it can be partially related to cheese chewiness. The obtained results suggest that the incorporation of inulin at the investigated concentrations can affect the chewability of giuncata cheese, despite the fact that this parameter is much more relevant for “hard cheese” with respect to “soft cheese” as giuncata. The positive area or stiffness is related to the area under the force vs. displacement plots, and it is a measure of the resistance to the penetration of the probe inside the cheese, having a similar meaning in terms of texture properties as the compression force. Adhesiveness, instead, is related to the work necessary to detach the material, such as cheese, from the testing probe. Being the values among the two tested giuncata, it can be supposed that the two cheeses have a similar sliceability.

#### 3.3.4. Water Content and Thermal Analysis

**Figure 6:**
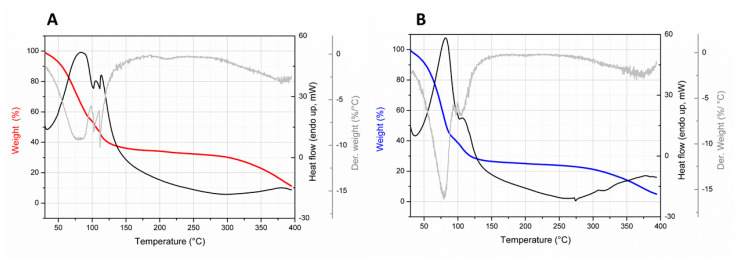
Weight (%), its derivative and heat flow (mW) vs. temperature plots for control giuncata cheese (Giuncata CTR) (**A**) and inulin-fortified giuncata (Giuncata IN) (**B**) as obtained from thermogravimetric analysis (TGA).

The water content was firstly determined by using a thermobalance and turned out to be 63.98 ± 1.82% for Giuncata CTR and 69.16 ± 0.39% for Giuncata IN, underlining that the presence of inulin increases the amount of water in the cheese. These results were confirmed by TGA analysis, showing a decrease in the sample weight for both samples between 64 and 72% (specifically, 64.76 ± 0.37% for Giuncata CTR and 71.61 ± 0.99% for Giuncata IN), related to the loss of water before 150 °C. The TGA technique was also able to discriminate between bound water and free water in the cheese since two or three slopes in the weight (%) trace can be recognized (Figure 6), one of them below 100 °C related to free water and the second and third one between 100 °C and 150 °C related to bound water [40]. This is also confirmed by the derivative of the weight signal, showing two or three minima at the temperatures at which the loss of free and bounded water is faster. The slight differences observed between the control and the samples could be attributed to a different kinetics of the water release from the cheese matrix [41]. This analysis also underlines that, as for the other soft cheese, the amount of free water in giuncata is much higher than the amount of bound water, being in the range of 68–72% with respect to the total water content. The endothermic transitions, evidenced from the heat flow signals (DTA analysis), having maxima corresponding to the minima in the derivate weight signals, confirm the release and evaporation of water from giuncata cheese occurring at temperatures at which the sample weight decreases. The degradation of the residual components of both giuncata cheese occurs at temperatures above 300 °C, at which the weight (%) decreases down to 10%.

## 4. Conclusions

This study led to the development of a new functional Italian “giuncata” cheese enriched with inulin. The fortification process achieved an inulin concentration of ~4% *w*/*w* in the fresh cheese, thereby claiming this dairy food product as a “source of dietary fiber” (inulin > 3 g/100 g according to European regulations on food). The addition of inulin determined some changes in the physical-chemical and mechanical properties, without altering the general appearance of the cheese. Only a little effect on the color, which becomes lighter, and a slight fat reduction have been observed in the inulin-fortified cheese with the respect to the control. A slightly “softening effect” was exerted by the incorporation of inulin in giuncata, as observed from rheological and mechanical analyses on cheese. Overall, these results demonstrate the feasibility of producing giuncata cheese as a functional dairy product, having possible benefits on human health such as the modulation of the composition of colonic microbiota, thanks to the prebiotic effect of inulin. Therefore, the results of this study can be supportive for an industrial scale-up in the production of functional cheese.

## Figures and Tables

**Table 1:** Concentrations of inulin in giuncata cheese at different fortification levels (10 g or 15 g of inulin added to milk).

Inulin Added to Milk (g)	Milk (g)	Produced Cheese (g)	Concentration of Inulin (g/100 g)
**10**	500	137.1 ± 12.0 ^a^	3.6 ± 0.3 ^a^
**15**	500	122.6 ± 4.5 ^b^	4.0 ± 0.1 ^b^

^a,b^ Values with different uppercase letters are significantly different (*p* < 0.05).

**Table 2:** Comparison of the chemical–physical characteristics (pH, Color, Fats percentage) between functionalized (Giuncata IN) and conventional (Giuncata CTR) cheese.

	Giuncata IN	Giuncata CTR
pH	6.6 ± 0.0 ^a^	6.5 ± 0.0 ^a^
Color		
- L*	95.6 ± 0.6 ^a^	93.9 ± 0.2 ^b^
- a*	−2.0 ± 0.3 ^a^	−1.8 ± 0.2 ^a^
- b*	7.5 ± 0.6 ^a^	7.8 ± 0.7 ^b^
Fats (Soxhlet extraction) Fats (Folch extraction)	11.72 ± 0.6% ^a^ 11.91± 0.2% ^a^	12.28 ± 0.7% ^b^ 12.52± 0.3% ^b^

^a,b^ Values with different uppercase letters are significantly different (*p* < 0.05).

**Table 3:** Textural parameter obtained from compression analysis of control giuncata cheese (Giuncata CTR) and inulin-fortified giuncata (Giuncata IN): Positive F_max_ “Compressive Force” (N), Negative Fmax (N), Positive area “Stiffness” (N mm), and Negative area “Adhesiveness” (N mm).

	Positive F_max_ “Compressive Force” (N)	Negative F_max_ (N)	Positive Area “Stiffness” (N mm)	Negative Area “Adhesiveness” (N mm)
Giuncata CTR	1.055 ± 0.291 ^a^	0.384 ± 0.083 ^a^	10.03 ± 3.24 ^a^	3.88 ± 1.43 ^a^
Giuncata IN	0.611 ± 0.127 ^b^	0.296 ± 0.041 ^b^	6.57 ± 1.10 ^b^	3.39 ± 0.46 ^a^

^a,b^ Values with different uppercase letters are significantly different (*p* < 0.05).

## Data Availability

The data used to support the findings of this study can be made available by the corresponding author upon request.

## Supplementary Materials

The following supporting information can be downloaded at: https://www.mdpi.com/article/10.3390/foods12183499/s1, Figure S1: (A) Sample of enriched cheese and Carrez reagents before clarification. (B) Extraction of inulin after clarification with Carrez reagents; Figure S2: Inulin extraction procedure from giuncata cheese; Table S1: Repeatability of the analytical method.
