# Supplementary material for: Inulin Functionalized “Giuncata” Cheese as a Source of Prebiotic Fibers
Source: Foods. 2023 Sep 20;12(18):3499. doi: 10.3390/foods12183499 (PMC10528394; doi:10.3390/foods12183499)
Supplement: Supplementary file 1 [file foods-12-03499-s001.zip › foods-2623120-supplementary.pdf]

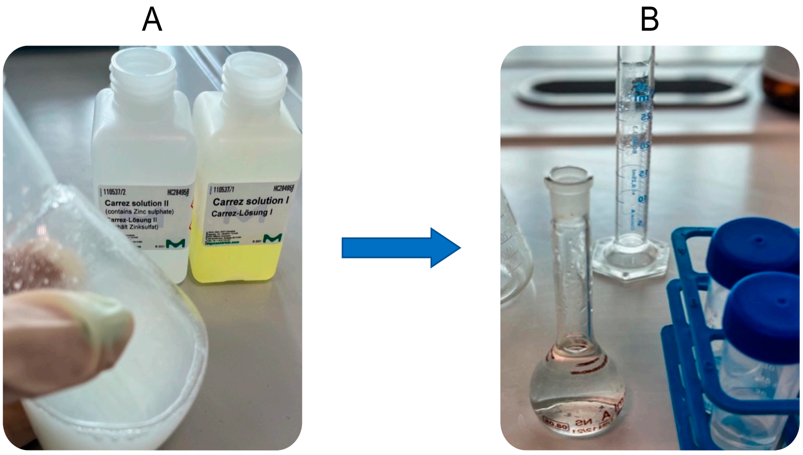

Figure S1. (**A**) Sample of enriched cheese and Carrez reagents before clarification. (**B**) Extraction of inulin after clarification with Carrez reagents.

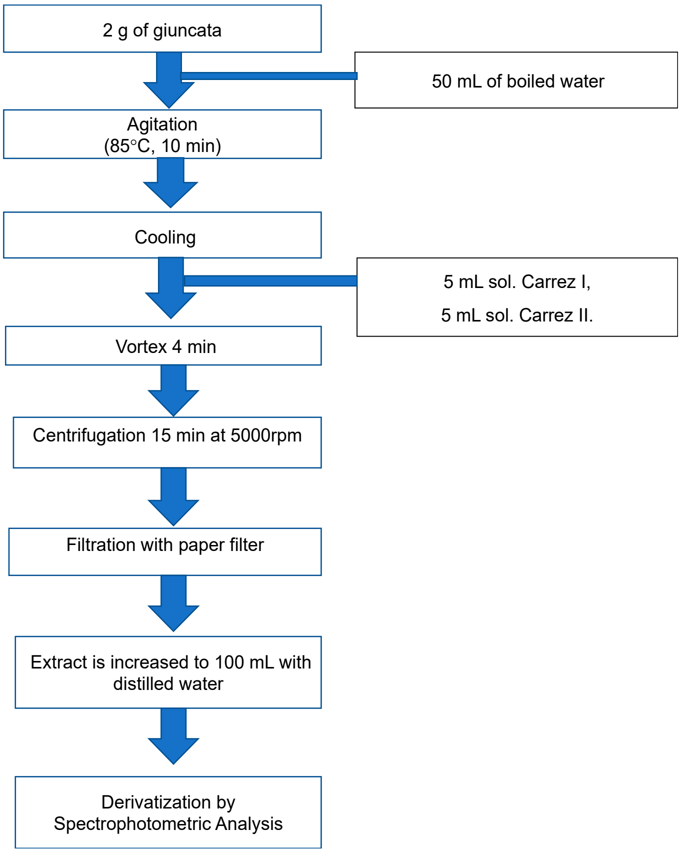

Figure S2. Inulin extraction procedure from giuncata cheese.

Table S1. Repeatability of the analytical method.

**Intraday**			**Intraday**		
Abs	Media	RSD%	Abs	Media	RSD%
0.1843	0.1843	0.2	0.1895	0.1885	0.7
0.1846			0.1885		
0.1841			0.1875		
